# The Role of Sports in Building Resilience: A Machine Learning Approach to the Psychological Effects of the COVID-19 Pandemic on Children and Adolescents

**DOI:** 10.3390/sports13020037

**Published:** 2025-02-03

**Authors:** Giulia Di Martino, Carlo della Valle, Alessandra di Cagno, Giovanni Fiorilli, Giuseppe Calcagno, Daniele Conte

**Affiliations:** 1 Department of Medicine and Health Sciences, University of Molise, 86100 Campobasso, Italy; giulia.dimartino21@gmail.com (G.D.M.); carlo.dellavalle@univr.it (C.d.V.); giuseppe.calcagno@unimol.it (G.C.); 2 Department of Neurosciences, Biomedicine and Movement, University of Verona, 37314 Verona, Italy; 3 Department of Movement, Human and Health Sciences, University of Rome “Foro Italico”, 00135 Rome, Italy; daniele.conte@uniroma4.it; 4 Department of Human Sciences, Guglielmo Marconi University, 00193 Rome, Italy

**Keywords:** pandemic, COVID-19, sports, psychological distress, adaptation process, anxiety, depression, resilience, adolescent, child, surveys and questionnaires

## Abstract

(1) Background: This study evaluated whether physical activity and sports serve as a protective factor in mitigating COVID-19 pandemic long-term consequences. (2) Methods: A survey of 1702 participants (8–18 years) used the Impact of Event Scale (IES), Generalized Anxiety Disorder Scale (GAD-7), and Center for Epidemiological Studies Depression Scale for Children (CES-DC). Participants were categorized based on their engagement in sports, cultural activities, or sedentary behaviors. Statistical analysis included non-parametric tests, CHAID models, and clustering. (3) Results: In total, 65.5% of participants experienced minimal to mild anxiety, and 34.5% reported moderate to severe anxiety. The main predictor of depression is the sample age, followed by the training experience. Depressive symptoms were identified in 71.3% of participants (75.7% adolescents; 64% children). Adolescents with longer training experience (67.6%) showed lower depression compared to those with shorter experience (77.2%). For post-traumatic stress, 38% of children and 46% of adolescents exceeded cut-off scores for significant symptoms, with age and training level identified as key predictors. Among children, sport experience with low weekly frequency was associated with the lowest depression rates (59.5%). (4) Conclusions: Four years after the pandemic, a high percentage of anxiety and depression disorders is observed in the youth population, particularly among adolescents. Sports appear to slightly alleviate this serious condition.

## 1. Introduction

Four years after its onset, the COVID-19 pandemic continues to raise significant concerns about the long-term consequences of the disease, leading the scientific community to investigate the complexities of the post-COVID clinical landscape. A great number of studies have revealed persistent symptoms extending beyond acute manifestations [1,2]. Among these, anxiety and depression emerge as significant issues requiring a thorough understanding and effective management strategies [3]. The current focus is now directed towards understanding the persistence of these effects among children and adolescents, addressing their specific needs [4,5].

Engagement in activities that stimulate children’s commitment and skills helps in overcoming these issues, especially when performed in groups and properly guided by a skilled operator. By cultural activities, we mean workshop, theatrical, and musical activities. Physical and sport activities have been recognized as a key element in promoting overall health and well-being, and psychological resilience in mitigating post-COVID psychological symptoms [6]. Sport performance is associated with self-esteem, emotional stability, and self-efficacy, enhancing individual proficiency in physical activities and strengthening resilience in managing daily difficulties [7]. Furthermore, sport and physical activity create the opportunity for social interactions, collaboration, and positive relationships [8]. Despite widespread recognition of its role in promoting physical health and equipping individuals with cognitive and emotional tools to manage stress, physical activity (PA) is also essential for preventing domestic isolation, mitigating technology overuse, and promoting healthier development in the younger population [9].

Addressing the connection between PA and post-COVID mental health is crucial for developing personalized preventive and therapeutic strategies tailored to age and previous difficulties.

The study aims to evaluate the persistence of psychological distress such as anxiety, depression, and post-traumatic stress disorders, across different age groups and to determine whether extracurricular cultural and physical and sport activities can provide resilience strategies in the young population. Unlike previous studies, this research employs an innovative decision-making approach using decision trees to identify the most efficient key predictors of these psychological outcomes.

## 2. Materials and Methods

### 2.1. Study Design

A cognitive survey was administered after 4 years of the COVID-19 pandemic (January 2024–June 2024) to evaluate the long-term psychological effects of the pandemic on children and adolescents, focusing on symptoms such as depression, anxiety, and stress. The study also aims to identify whether sports activity contributes to enhancing resilience and mitigating these psychological outcomes.

### 2.2. Participants

A total of 1702 children and adolescents were recruited through a snowball sampling strategy using an online survey platform (Google Form) or via paper questionnaires administered at school complexes. The inclusion criteria were (a) aged between 8 and 18 years; (b) affiliation with sports clubs; (c) practicing cultural or amateur sports activities; (d) sedentary individuals. The study participants were distributed as follows: by type of activity (athletes, individuals practicing cultural activities, those practicing both sports and cultural activities, sedentary individuals), and by age group (8–12 years and 13–18 years).

Sporting children and adolescents were classified based on individual or team sports. According to the competitive level, athletes were divided into competitive or amateur categories. Athletes were grouped into different categories based on workload (minutes per week).

The study was designed and conducted in accordance with the Declaration of Helsinki and approved by the University Research Committee (CAR-IRB) of the University of Rome “Foro Italico” (CAR 178/2023).

### 2.3. Procedures

To assess the potential impact on quality of life, levels of depression, anxiety, and perceived stress four years after the COVID-19 pandemic, a cognitive survey was conducted.

The questionnaires were administered in Italy using two methods. The first method involved surveying an online platform (Google Forms). Invitations were sent to National Sports Federations, Sports Promotion Entities, and Cultural Centers. An informative letter was included, explaining the purpose of the research and ensuring confidentiality and anonymity. Personal data were collected anonymously through the use of a personal security code. Informed consent was obtained electronically. Adult participants (aged 18) completed the consent form after reading and accepting the study description. For minors, consent was provided by parents or guardians.

The second survey was conducted in paper format in school settings during curricular hours. Participation was promoted by school principals, who informed families via a circular that provided an overview of the project. After reviewing the document, parents submitted the informed consent forms to the school, which were subsequently delivered to the research team.

In the first section, the survey assesses sociodemographic factors such as age, gender, “type of activity” (sport, cultural activities, both sport and cultural activities, no sport), “training and cultural activities experience” (years), and “physical activity level” (time × day × week).

The second section evaluates the quality of life, levels of depression, anxiety, and psychological distress four years after the COVID-19 pandemic, using three different questionnaires: the Impact of Event Scale (IES-8 and IES-15), Generalized Anxiety Disorder Scale (GAD-7), and Center for Epidemiological Studies Depression Scale for Children (CES-DC).

### 2.4. Screening Questionnaire

#### 2.4.1. Impact of Event Scale

The Impact of Event Scale (IES) questionnaire was used to assess post-traumatic stress disorder (PTSD) in children and adolescents, specifically regarding the psychological impact of the COVID-19 pandemic. This includes factors such as lockdown measures, social isolation, and trauma related to the virus. The goal is to identify the effects of these stressors and evaluate the protective role of physical activity.

Two different databases were used based on the age of the participants: children (aged from 8 to 12 years) underwent the IES-8 [10], while adolescents (aged 13 to 18 years) underwent the IES-15 [11]. Both the IES-8 and IES-15 questionnaires include two subscales to evaluate the symptoms of intrusion (INT) and avoidance (AV). The subscale scores highlight the frequency of specific behaviors. The INT subscale measures the recurrence of trauma-related thoughts, images, dreams, and emotions, while the AV subscale evaluates attempts to suppress those thoughts and feelings. AV also includes elements of emotional numbness and dissociation, which represent an active defensive response.

The IES-8 and IES-15 questionnaires include 8 and 15 items, respectively, and use a Likert scale that ranges from 0 (not at all) to 5 (often).

The IES-8 [10], designed for children aged 8 to 12, shows a strong correlation with the full version (IES-R) and has a cut-off score of 17 or above to identify individuals experiencing stress. The scale showed a strong overall reliability, with a coefficient of 0.84. The INT and AV subscales have achieved reliability values of 0.91 and 0.83, respectively. Furthermore, the IES-8 showed high internal consistency, with a Cronbach’s alpha of 0.78 [12]. It has been previously used to assess children’s reactions to potentially traumatic events.

The IES-15 [11] is designed for participants aged 13 to 18. A total score of 30 or above indicates a significant stress response syndrome. The subscale scores for INT and AV help assess the likelihood of these behavioral patterns. The IES-15 shows strong test-retest reliability, with a range of 0.79 to 0.89, and showed satisfactory internal consistency (Cronbach’s α = 0.78 to 0.82) [13].

Both versions used in this study have been validated in Italy [14,15].

#### 2.4.2. Generalized Anxiety Disorder Scale

The Generalized Anxiety Disorder Scale (GAD-7) [16], was used to assess the presence and severity of generalized anxiety disorder symptoms in the past two weeks. The GAD-7 includes 7 items, each one with a Likert rating scale from 0 to 3 on a 4-point Likert scale (0 = Not at all; 1 = several days; 2 = more than half the days; 3 = nearly every day). The total score ranges from 0 to 21, with higher scores indicating greater anxiety severity: 0–4 = minimal anxiety; 5–9 = mild anxiety; 10–14 = moderate anxiety; 15–21 = severe anxiety. A score of 10 or higher is generally considered the cutoff for identifying clinically significant anxiety, which requires further evaluation or intervention by a healthcare professional [17]. Furthermore, the GAD-7 demonstrated high reliability, with a Cronbach’s alpha of 0.895 [18].

#### 2.4.3. Center for Epidemiological Studies Depression Scale for Children

The Center for Epidemiological Studies Depression Scale for Children (CES-DC) was used to evaluate depressive symptoms in children and adolescents. The CES-DC consists of 20 items that assess the presence and severity of depressive symptoms experienced during the past week. Each item was rated on a 4-point Likert scale, ranging from 0 (“not at all”) to 3 (“a lot”). The total score ranges from 0 to 60, with a cut-off score of 15 or higher generally indicating the possible presence of clinically significant depression. The CES-DC has strong internal consistency, as reflected by Cronbach’s alpha values of 0.85 [19].

### 2.5. Statistical Analysis

Descriptive statistics were calculated using percentages (%) for categorical data, while mean, SD, median and inter-quartile ranges were calculated for continuous data.

Furthermore, separated two-step cluster analyses were applied for the two continuous variables (i.e., age and normalized training experience) when considering the samples of GAD and CES-DC, while the same cluster analysis approach was used for normalized training experience only when considering IES-15 and IES-8. The two step cluster analysis was calculated using a Schwarz’s Bayesian Criterion (BIC) and including a pre-determined number of clusters (i.e., 3). For the cluster formation, the analysis of the Silhouette measure of cohesion and separation was also performed with values interpreted as 0.0–0.2, poor; 0.2–0.8, fair; and 0.8–1, high [20]; with all cluster analyses revealing fair to high values.

The Shapiro–Wilk test was applied to assess the normal distribution of the dependent variables (i.e., the total scores of GAD, CES-DC, IES-15, and IES-8, the subscales of intrusion and avoidance for IES-15 and IES-8), showing data were non-normally distributed. Therefore, to assess the differences or the variables cultural activity and sex, a Mann–Whitney U test was applied for each dependent variable, while Kruskal–Wallis tests were used to assess the differences for physical activity levels, cluster age, cluster normalized training experience, and type of activity. In case of statistical significance difference, a post hoc analysis using the Mann–Whitney U test with Bonferroni correction was applied.

Successively, separate exhaustive CHAID decision trees using a maximum of three levels about the tree depth, parent node = 100, child node = 50, and including the Pearson’s chi-square statistics to split the nodes were used as the machine learning approach. Specifically, total SRTS values for GAD (minimal/mild anxiety vs. moderate/severe anxiety), cut-off values from CES-DC (depressed vs. non-depressed), and IES-8 and IES-15 were used as dependent variables and sex (male vs. female), age (i.e., 3 clusters), physical activity level (≤120 min, 121–270 min, 271–480 min, >480 min), type of activity (sport, cultural activity, both or none), normalized training experience (i.e., 3 clusters), and cultural activity (yes vs. no) were included in the model as independent variables. Cross-validation was used to assess the robustness of the created tree using 10 as the number of sample folds. The predictive ability of each decision tree model was assessed using the resubstitution method.

All analyses were run using SPSS (IBM, v.29.0, Chicago, IL, USA) and the significance level for the non-parametric tests and the splitting nodes was set at <0.05.

## 3. Results

The sports participants who completed the questionnaire practiced 45.80% individual sports and 54.20% team sports. The main sports activities practiced were soccer, fencing, rhythmic gymnastics, basketball, dance, swimming, and tennis.

### 3.1. Cluster Analyses

#### 3.1.1. GAD and CES-DC

According to the analysis for GAD and CES-DC, the three clusters for age had the following mean ± SD: 10.7 ± 1.1 years, 14.1 ± 0.7 years, and 17.4 ± 1.1 years. The normalized training experience showed the following mean ± SD for the three clusters, respectively: 0.05 ± 0.04 AU (low normalized training experience), 0.26 ± 0.07 AU (moderate normalized training experience), 0.54 ± 0.11 AU (high normalized training experience).

#### 3.1.2. IES-15 and IES-8

Regarding the analysis of the IES-15, the three clusters for normalized training experience had the following mean ± SD: 0.04 ± 0.04 AU, 0.23 ± 0.05 AU, 0.46 ± 0.10 AU. Moreover, the normalized training experience showed the following mean ± SD for the three clusters when considering IES-8: 0.11 ± 0.08 AU, 0.66 ± 0.73 AU, 0.39 ± 0.07 AU.

### 3.2. Traditional Non-Parametric Approach

#### 3.2.1. GAD

No differences in sex, cultural activity, PA level, and type of activity were found for the GAD total score, while a significant difference was found for cluster age and normalized training experience (*p* < 0.001). Post hoc analyses of cluster age revealed that younger participants (i.e., 10.7 ± 1.1 years) had lower GAD scores compared to older participants grouped in cluster 2 (*p* < 0.001) and cluster 3 (*p* < 0.001). Regarding the normalized training experience, higher values of GAD total score were found in participants reporting a lower normalized training experience (*p* < 0.001) and medium normalized training experience (*p* < 0.001) compared to high normalized training experience. The results are shown in Table 1.

#### 3.2.2. CES-DC

No differences in total score for sex, cultural activity, and PA levels (*p* > 0.05) were found. Differently, differences were evident in cluster age and normalized training experience (*p* < 0.001) and type of activity (*p* = 0.036). Post hoc analyses of age revealed that younger participants (i.e., 10.7 ± 1.1 years) had lower CES-DC values compared to older participants grouped in Cluster 2 (*p* < 0.001) and Cluster 3 (*p* < 0.001), while no differences were evident between Cluster 2 and Cluster 3 (*p* > 0.05). Considering normalized training experience, post hoc analyses showed that participants with a high normalized training experience had lower CES total score compared to participants with low (*p* < 0.001) and medium (*p* < 0.001) normalized training experience. The only post hoc difference found in type of activity included lower CES total score values for sport compared to no activity (*p* < 0.05). The results are shown in Table 1.

#### 3.2.3. IES-15

No effects were evident for cultural activity, normalized training experience, PA levels, and type of activity for any considered dependent variable (*p* > 0.05). Similarly, no differences were evident for sexes when considering total score and intrusion mean (*p* > 0.05), while a difference was evident for avoidance mean (*p* = 0.027). The results are reported in Table 2.

#### 3.2.4. IES-8

Higher values for the female participants compared to the male participants were evident for IES-8 total score (*p* = 0.014, ES: Z = −2.466) and mean intrusion (*p* = 0.040; ES: Z = −2.059). No effect was evident for type of activity, physical activity level, and cultural activity (*p* > 0.05). Moreover, an effect was found for normalized training experience in total score (*p* = 0.039) and mean intrusion (*p* = 0.011). Post hoc analyses showed that low normalized training experience had a lower total score (*p* < 0.05 Z = −2.542) and mean intrusion (*p* < 0.05 Z = −2.935) compared to medium normalized training experience. The results are shown in Table 3.

### 3.3. Decision Trees Results

#### 3.3.1. GAD

**Figure 1:**
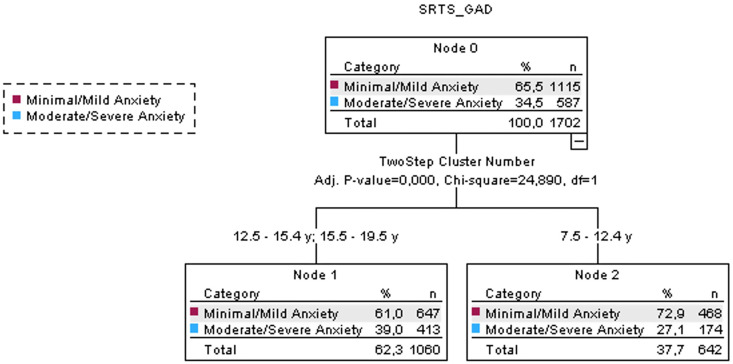
Results with decision tree model of Generalized Anxiety Disorder Scale (GAD): age has been identified as the main predictor of anxiety levels (Generalized Anxiety Disorder Scale).

The analysis of anxiety revealed that most of the participants showed a minimal/mild anxiety (65.5%), while moderate/severe anxiety represented 34.5% of the sample considered. The decision tree analysis showed age as the only predictor of anxiety with participants aged >12.5 y documenting a moderate/severe anxiety of 39% (Figure 1). The decision tree model reported an error rate of 34%.

#### 3.3.2. CES-DC

**Figure 2:**
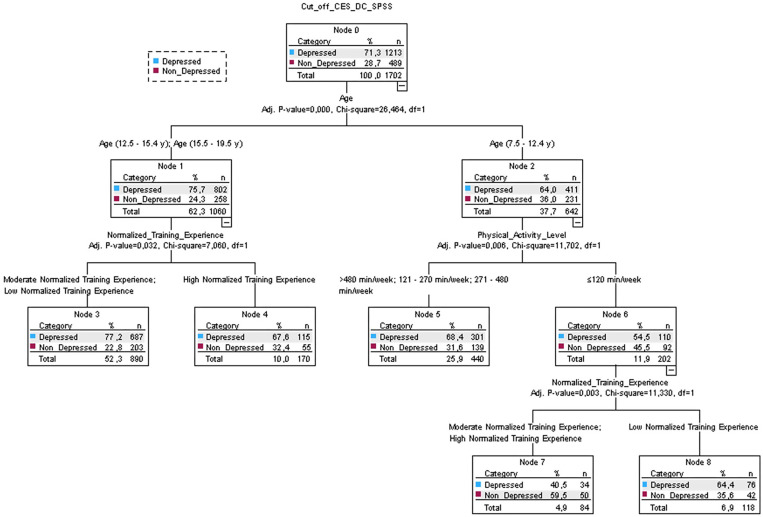
Results with decision tree model on the questionnaire “Center for Epidemiological Studies Depression Scale for Children (CES-DC)”: age was the primary predictor for depression. Participants were divided into Node 1 and Node 2: for Node 1, the second predictor for age categories 12.5–15.4 y and 15.5–19.5 y was the training experience, while for Node 2, the main predictor was physical activity level.

Considering depressions (CES-DC), 71.3% of the participants showed depressive symptoms (Figure 2). The main predictor is age followed by normalized training experience and PA level. In particular, the highest likelihood of depressive symptoms is present in participants with ≥12.5 y and with moderate-to-low normalized training experience (77.2%). The only case in which participants showed non depression symptoms (59.5%) is shown following the right path of the decision tree, including kids (<12.4 y) with low PA levels (≤120 min/week) but with Moderate-to-High normalized training experience. This decision tree model reported an error rate of 28%.

#### 3.3.3. IES 15

For this questionnaire, no variable was retained in the model and it was not possible to build the decision tree which remained only with the root node (moderate/severe 24.6% [n = 257] and normal/mild 75.4% [n = 786]).

#### 3.3.4. IES-8

**Figure 3:**
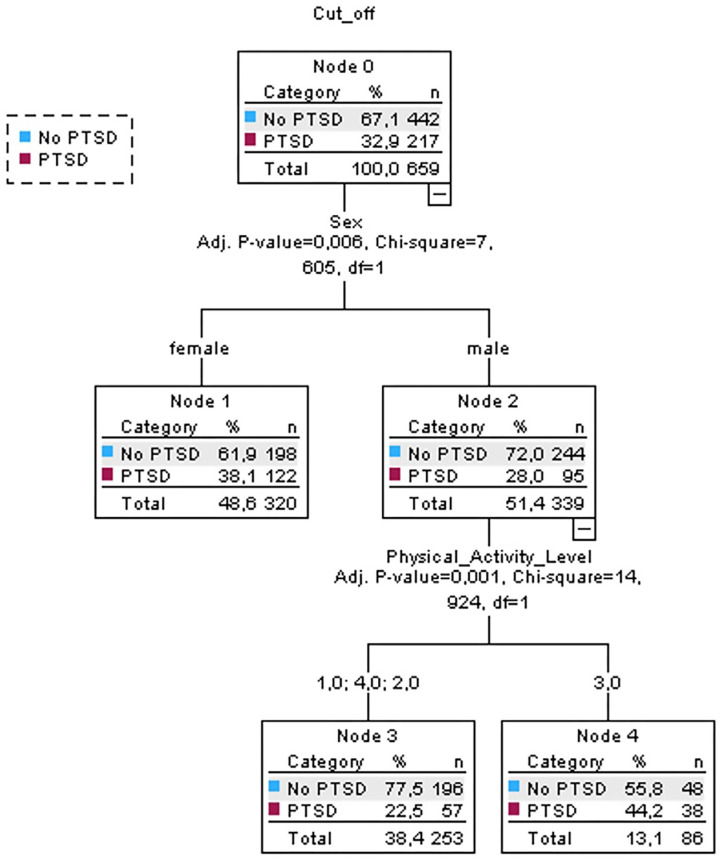
Results with decision tree model of Impact of Event Scale-8 (IES-8): sex was the primary prediction of post-traumatic stress disorder. Participants were divided into Node 1 and Node 2: for Node 2, physical activity level was the main predictor.

Considering IES-8, 32.9% of the participants showed PTSD symptoms (Figure 3). The main independent variable retained in the model is sex with male participants showing lower percentage of participants with PTSD (28%) compared to female participants (38.1%). Considering males only, PA level was the other variable retained in the model with limited (≤120 min, 121–270 min) or very high (>480 min) PA levels showing the highest values of no PTDS symptoms. Differently, participants reporting 271–480 min of PA per week showed lower percentages (55.8%) of no PTSD symptoms. This decision tree model reported an error rate of 33%.

## 4. Discussion

This study aimed to investigate trends in psychological issues among children and adolescents and to evaluate whether extracurricular cultural and sports activities played a protective role in mitigating the long-term effects of the COVID-19 pandemic. The findings confirmed that anxiety, depression, and post-traumatic stress disorder (PTSD) remain significant concerns, particularly among adolescents, as a significant consequence, among others, of prolonged isolation [21,22].

Participation in cultural activities proved neutral in addressing psychological issues. In contrast, physical activity demonstrated potential in mitigating these adverse effects by enhancing self-esteem and promoting social support, probably due to sport participation, with differences for age, gender, and the level of sports involvement. It was expected that sport participation leads to psychological resilience, providing young people with cognitive as well as emotional tools to counteract stressors [23].

A central aspect of this study concerns the use of an innovative approach using a decision tree that, assessing differences in the key variables, detected a risk profile for participants.

Adolescents are the most impacted age group, exhibiting higher levels of anxiety and depressive symptoms compared to children, with no significant difference observed between Cluster 2 (14.1 ± 0.7 age years) and Cluster 3 (17.4 ± 1.1 age years) [24]. The physical and sports activity intervention should therefore particularly involve adolescents, proposing high-impact activities in terms of intensity and duration, to reduce anxiety levels in this group. Decision tree models further highlighted age as the primary predictive factor for anxiety (ranged from moderate to high), while for depression, key predictors included age, normalized sports training experience, and physical activity level, particularly among adolescents. These adolescents were children during the pandemic, and the loss of daily routines likely contributed to heightened anxiety [25]. This is consistent with the understanding that uncertainty—amplified by the disruption of school, social, and sports activities—acts as a key contributor to anxiety [26].

Regarding depression, it should be taken into consideration that adolescents have been more significantly impacted by reduced peer interactions and increased demands for personal responsibility, such as self-directed learning [27]. High-intensity training also proves effective in counteracting depressive symptoms, taking into consideration that adolescents are more affected than children (age as the main predictor). Furthermore, the results highlighted that engaging in sports activities with others could be a solution to counter depression, even though no significant differences were found between team sports and individual sports in the obtained results. Research on psychological development during childhood and adolescence indicates that adolescents are particularly susceptible to social stressors like isolation, loneliness, and boredom and, that symptoms of affective disorders increase markedly during this period [28].

The social dimension of sports and PA helps build meaningful connections, providing an emotional support network for adolescents, enhancing adaptive coping strategies, and supporting emotional well-being. The analysis demonstrated that a greater sports experience, in terms of both duration and intensity, is associated with lower levels of depression and could be a key factor in protecting individuals from psychological issues. Children were less impacted by the pandemic’s mental health effects, benefiting from stable home environments and supportive parenting, though high parental stress could still lead to maladaptive responses [29]. However, for children under the age of 12 years, the weekly training load should not be excessively high, suggesting the need for a balanced approach to training [25].

Considering PTSD symptoms, higher levels of PA, ranging from high to very high, were associated with reduced PTSD symptoms in children, while no significant effects were observed in adolescents. Despite gender not being identified as a key factor influencing anxiety and depression, the only significant sex difference was identified in PTSD symptoms in children, with a higher percentage of stress disorders reported in females compared to males. The imbalance can be attributed both to biological and sociocultural differences between males and females since this difference appears in children [30]. Nevertheless, the results that gender differences in managing stress were found only in children could be explained as follows: adolescents may have a background in physical activity and sports that could have provided them with greater protection, with no gender differences, compared to children [31].

Sports train young people to face continuous challenges, thereby enhancing their resilience skills [32].

The study, however, highlights some limitations: although only a few questionnaires were collected through the snowball sampling method, as the majority were gathered in person during school lessons, we highlight that no stratified random sampling was performed for this small group. Socioeconomic status and prior mental health conditions were not investigated, and this may have introduced bias in the interpretation of the results.

## 5. Conclusions

This approach underscores the added value of advanced analytical techniques for informing more targeted intervention strategies.

The results of this study highlighted the significant presence of psychological issues among children and adolescents. It is also confirmed in this study that physical activity and sports have proven to be effective strategies in mitigating the long-term psychological impacts of the COVID-19 pandemic.

Promoting regular and balanced physical activity, tailored to age and developmental stages, could be an effective preventive strategy against anxiety, depression, and PTSD symptoms, fostering coping strategies.

Adolescents, who have shown higher vulnerability to psychological distress, particularly require targeted interventions such as activities that emphasize team-based participation and peer interaction. Family involvement also could play a crucial role, especially for younger children. The results of the present study could encourage public health policies and educational programs by emphasizing the importance of physical and school-based activities. Specifically, organizing sports programs within school curricula during school hours could help improve students’ post-COVID stress levels.

## Figures and Tables

**Table 1:** Results with traditional non-parametric approach of Generalized Anxiety Disorder Scale (GAD) and Center for Epidemiological Studies Depression Scale for Children (CES-DC).

Variable		Total Score GAD		Total Score CES-DC	
		Mean (SD)	Median (IQR)	Mean (SD)	Median (IQR)
Sex	Male	7.6 (4.8)	7.0 (7.0)	21.0 (9.3)	20.0 (14.0)
	Female	7.9 (5.1)	7.0 (7.0)	22.1 (10.1)	21.0 (15.0)
Type of activity	Sport	7.7 (5.0)	7.0 (7.0)	21.4 (9.6)	20.0 (14.0)
	Cultural activity	7.5 (5.4)	6.0 (6.7)	21.4 (11.3)	19.5 (20.0)
	Both sport and cultural activity	7.7 (4.8)	7.0 (7.0)	21.0 (9.2)	20.0 (12.0)
	No activity	8.2 (5.2)	8.0 (8.0)	23.6 (10.6)	23.0 (17.0)
Physical activity level	≤120 min/week	7.8 (5.1)	7.0 (7.0)	21.8 (10.6)	20.0 (16.0)
	121–270 min/week	7.4 (4.7)	7.0 (6.0)	21.4 (9.3)	20.0 (13.0)
	271–480 min/week	8.0 (4.9)	7.0 (7.0)	21.5 (9.4)	21.0 (13.0)
	>480 min/week	8.0 (5.1)	7.0 (7.0)	21.6 (9.5)	21.0 (14.0)
Cultural activity	Yes	7.4 (5.0)	7.0 (7.0)	20.5 (9.8)	19.0 (13.0)
	No	7.9 (4.7)	7.0 (7.0)	21.7 (9.0)	21.0 (14.0)
Normalized training experience	Low	8.1 (5.1)	7.0 (7.0)	23.0 (10.1)	22.0 (15.0)
	Medium	8.2 (5.1)	7.0 (8.0)	21.6 (9.6)	21.0 (14.0)
	High	6.8 (4.6)	6.0 (7.0)	19.5 (9.2)	19.0 (14.0)
Age	10.7 (1.1) y	6.6 (4.4)	6.0 (7.0)	19.7 (9.1)	19.0 (14.0)
	14.1 (0.7) y	8.6 (5.2)	8.0 (8.0)	22.7 (10.1)	21.0 (15.0)
	17.4 (1.1) y	8.4 (5.1)	8.0 (7.0)	22.8 (9.8)	22.0 (14.0)

Legend: SD: Stantard Deviation; IQR: Interquartile Range; GAD: Generalized Anxiety Disorder Scale; CES: Center for Epidemiological Studies Depression Scale for Children.

**Table 2:** Results with traditional non-parametric approach of Impact of Event Scale-15 (IES-15).

Variable		Total Score		Mean Intrusion		Mean Avoidance	
		Mean (SD)	Median (IQR)	Mean (SD)	Median (IQR)	Mean (SD)	Median (IQR)
Sex	Male	16.7 (11.0)	15.0 (16.0)	1.0 (0.9)	0.9 (0.9)	1.6 (1.1)	1.6 (1.6)
	Female	18.1 (12.0)	16.9 (17.0)	1.1 (0.9)	0.9 (1.0)	1.8 (1.2)	1.6 (1.4)
Type of activity	Sport	17.5 (11.7)	16.0 (18.0)	1.1 (0.9)	0.9 (1.4)	1.7 (1.2)	1.6 (1.6)
	Cultural activity	N/A	N/A	N/A	N/A	N/A	N/A
	Both sport and cultural Activity	16.8 (11.5)	15.0 (15.8)	1.1 (0.9)	0.9 (1.3)	1.6 (1.2)	1.4 (1.6)
	No activity	17.3 (10.5)	17.0 (16.0)	1.1 (0.9)	1.0 (1.2)	1.6 (1.0)	1.6 (1.2)
Physical activity level	≤120 min/week	16.4 (11.3)	16.0 (15.0)	1.0 (0.9)	0.9 (1.1)	1.6 (1.1)	1.6 (1.4)
	121–270 min/week	18.6 (11.7)	18.0 (16.0)	1.1 (0.9)	1.0 (1.3)	1.8 (1.2)	1.6 (1.6)
	271–480 min/week	17.9 (11.3)	15.0 (16.3)	1.0 (0.9)	0.9 (1.2)	1.7 (1.1)	1.6 (1.6)
	>480 min/week	16.9 (11.7)	15.0 (19.0)	1.1 (0.9)	0.9 (1.4)	1.6 (1.2)	1.4 (1.6)
Cultural activity	Yes	15.7 (10.2)	14.5 (13.5)	1.0 (0.8)	0.9 (0.9)	1.5 (1.1)	1.3 (1.6)
	No	17.5 (11.6)	16.0 (17.0)	1.1 (0.9)	0.9 (1.4)	1.7 (1.2)	1.6 (1.6)
Normalized training experience	Low	17.7 (11.3)	17.5 (17.0)	1.1 (0.9)	1.0 (1.2)	1.7 (1.1)	1.6 (1.4)
	Medium	17.9 (11.8)	16.0 (17.0)	1.1 (0.9)	0.9 (1.3)	1.7 (1.2)	1.6 (1.6)
	High	16.1 (11.4)	14.0 (16.0)	1.0 (0.9)	0.7 (1.3)	1.5 (1.2)	1.3 (1.8)

Legend: SD: Standard Deviation; IQR: Interquartile Range; N/A: Not Available.

**Table 3:** Results with traditional non-parametric approach of Impact of Event Scale-8 (IES-8).

Variable		Total Score		Mean Intrusion		Mean Avoidance	
		Mean (SD)	Median (IQR)	Mean (SD)	Median (IQR)	Mean (SD)	Median (IQR)
Sex	Male	12.3 (7.6)	12.0 (10.5)	1.4 (1.0)	1.3 (1.5)	1.7 (1.4)	1.5 (2.3)
	Female	13.7 (7.6)	13.5 (11.0)	1.6 (1.0)	1.5 (1.5)	1.9 (1.4)	1.5 (2.3)
Type of activity	Sport	12.9 (7.4)	12.0 (11.0)	1.4 (1.0)	1.3 (1.5)	1.8 (1.4)	1.5 (2.4)
	Cultural Activity	14.3 (8.6)	12.0 (9.8)	2.0 (1.1)	2.3 (1.6)	1.6 (1.5)	1.5 (2.4)
	Both Sport and Cultural Activity	13.3 (8.7)	13.0 (13.3)	1.6 (1.1)	1.5 (2.0)	1.8 (1.5)	1.5 (2.8)
	No Activity	13.0 (6.7)	13.0 (9.0)	1.4 (1.0)	1.5 (1.4)	1.8 (1.3)	1.6 (1.8)
Physical activity level	≤120 min/week	13.2 (7.8)	13.0 (11.3)	1.4 (1.0)	1.3 (1.8)	1.9 (1.4)	1.8 (2.1)
	121–270 min/week	12.0 (7.4)	11.0 (12.0)	1.4 (1.0)	1.3 (1.8)	1.6 (1.3)	1.5 (1.8)
	271–480 min/week	14.2 (7.7)	14.5 (12.0)	1.6 (1.0)	1.5 (1.4)	1.9 (1.4)	1.5 (2.5)
	>480 min/week	12.7 (7.4)	12.0 (11.0)	1.4 (1.0)	1.5 (1.4)	1.8 (1.4)	1.5 (2.8)
Cultural activity	Yes	13.4 (8.7)	12.0 (13.0)	1.6 (1.1)	1.5 (1.9)	1.7 (1.5)	1.5 (2.6)
	No	12.9 (7.4)	13.0 (11.0)	1.4 (1.0)	1.3 (1.5)	1.8 (1.4)	1.5 (2.1)
Normalized training experience	Low	13.7 (7.4)	13.0 (10.5)	1.6 (1.0)	1.5 (1.5)	1.9 (1.3)	1.8 (2.0)
	Medium	12.0 (7.6)	11.0 (12.0)	1.3 (1.0)	1.3 (1.5)	1.7 (1.4)	1.5 (2.5)
	High	13.2 (8.2)	12.0 (12.0)	1.5 (1.0)	1.3 (1.3)	1.9 (1.5)	1.5 (2.8)

Legend: SD: Standard Deviation; IQR: Interquartile Range.

## Data Availability

The data are available by request to the corresponding author.
